# Diversity, abundance and activity of ammonia-oxidizing microorganisms in fine particulate matter

**DOI:** 10.1038/srep38785

**Published:** 2016-12-12

**Authors:** Jing-Feng Gao, Xiao-Yan Fan, Kai-Ling Pan, Hong-Yu Li, Li-Xin Sun

**Affiliations:** 1 College of Environmental and Energy Engineering, Beijing University of Technology, Beijing 100124, China

## Abstract

Increasing ammonia emissions could exacerbate air pollution caused by fine particulate matter (PM_2.5_). Therefore, it is of great importance to investigate ammonia oxidation in PM_2.5_. This study investigated the diversity, abundance and activity of ammonia oxidizing archaea (AOA), ammonia oxidizing bacteria (AOB) and complete ammonia oxidizers (Comammox) in PM_2.5_ collected in Beijing-Tianjin-Hebei megalopolis, China. *Nitrosopumilus* subcluster 5.2 was the most dominant AOA. *Nitrosospira multiformis* and *Nitrosomonas aestuarii* were the most dominant AOB. Comammox were present in the atmosphere, as revealed by the occurrence of *Candidatus* Nitrospira inopinata in PM_2.5_. The average cell numbers of AOA, AOB and *Ca.* N. inopinata were 2.82 × 10^4^, 4.65 × 10^3^ and 1.15 × 10^3^ cell m^−3^ air, respectively. The average maximum nitrification rate of PM_2.5_ was 0.14 μg (NH_4_^+^-N) [m^3^ air·h]^−1^. AOA might account for most of the ammonia oxidation, followed by Comammox, while AOB were responsible for a small part of ammonia oxidation. Statistical analyses showed that *Nitrososphaera* subcluster 4.1 was positively correlated with organic carbon concentration, and *Nitrosomonas eutropha* showed positive correlation with ammonia concentration. Overall, this study expanded our knowledge concerning AOA, AOB and Comammox in PM_2.5_ and pointed towards an important role of AOA and Comammox in ammonia oxidation in PM_2.5_.

Ammonia (NH_3_), the primary alkaline gas in atmosphere, contributes to the formation of fine particulate matter (PM_2.5_, aerodynamic diameter less than or equal to 2.5 μm). PM_2.5_, the main air pollutant in urban cities, can decrease visibility, modify radiation balance of the Earth, reduce lung function and increase pulmonary disease^1^. PM_2.5_ is a complex mixture of different inorganic and organic substances. Especially, a large part (20–80%) of the total PM_2.5_ mass is secondary inorganic aerosol (SIA)^2^, which is responsible for regional-scale pollution^3^. SIA is predominantly in the form of ammonium sulfate ((NH_4_)_2_SO_4_), ammonium bisulfate (NH_4_HSO_4_), and ammonium nitrate (NH_4_NO_3_)^4^. The formation of these aerosols is mainly due to neutralization of ammonia by sulphuric acid and nitric acid^4^. Therefore, controlling ammonia emissions will effectively reduce PM_2.5_ pollution.

Ammonia is also the most abundant reduced form of reactive nitrogen (Nr) in the atmosphere. Nr is highly mobile and widely distributed, the deposition of Nr can reduce the biodiversity and disturb the global biogeochemical cycling of nitrogen^5^. The air pollution caused by PM_2.5_ and Nr is in part closely linked to each other. The inorganic species of Nr are important precursors of the secondary inorganic ions of PM_2.5_^5^. Thus, ammonia plays an important role in air quality, human health, ecosystem health and global biogeochemical cycling of nitrogen. Rebalancing the global nitrogen cycle is the key to controlling Nr and PM_2.5_ pollutions.

Nitrification, microbial oxidation of ammonia to nitrate, is a central step of the global nitrogen cycle, which links the gain and loss of bioavailable nitrogen^6^. This process was thought to involve two separate steps: ammonia oxidation (NH_3_-NO_2_^−^) and nitrite oxidation (NO_2_^−^-NO_3_^−^). Ammonia oxidation is the rate-limiting step of nitrification. Before 2015, this process is considered to be primarily performed by two main distinct ammonia-oxidizing microorganisms (AOMs): ammonia-oxidizing bacteria (AOB) and ammonia oxidizing archaea (AOA). The phylogenetic distribution of AOB is limited to *Betaproteobacteria* (Beta-AOB) and *Gammaproteobacteria* (Gamma-AOB), and AOA fall within *Thaumarchaeota*. AOB fall into three major clusters: *Nitrosococcus*, *Nitrosomonas* and *Nitrosospira*^7^. AOA are classified into five major clusters: *Nitrososphaera*, *Nitrosopumilus*, *Nitrosotalea*, *Nitrosocaldus* and *Nitrososphaera* sister cluster^8^. Numerous surveys about the diversity, abundance and distribution of AOA and AOB have been undertaken in a variety of environments, such as sediments^9^, soils^10^, estuaries^11^ and wastewater treatment plants (WWTPs)^12^. Recently, the discovery of complete ammonia oxidizers (Comammox), i.e., bacteria within the genus *Nitrospira* that completely oxidize ammonia to nitrate, has significantly expanded our understanding of nitrification^13^,^14^. Until now, there are three Comammox enrichment cultures: *Candidatus* Nitrospira inopinata, *Candidatus* Nitrospira nitrosa and *Candidatus* Nitrospira nitrificans^13^,^14^. Comammox *amoA* gene sequences fall into two clades (clade A and clade B), and *Ca.* N. inopinata *amoA* gene is classified into Comammox *amoA* clade A^13^. The studies of van Kessel *et al*. and Daims *et al*. suggest that Comammox are widely distributed, including agricultural soils, freshwater environments, WWTPs and drinking water systems^13^,^14^. Overall, AOA, AOB and Comammox are widely distributed in nature and engineered environments. Moreover, AOA may easily outcompete AOB under oligotrophic conditions based on the physiological properties of the available AOA cultures^15^,^16^. Comammox may also have an advantage over AOA and AOB under substrate-limiting conditions, such as biofilms and microbial aggregates where the ammonia concentration is low^6^,^14^. Therefore, atmosphere may be another habitat for AOA and Comammox due to limited nutrient availability. However, very little is known about AOA, AOB and Comammox in the atmosphere, especially in PM_2.5_.

The occurrence and diversity of AOA in the coarse particulate matter (>3 μm) samples collected in Mainz, Germany are reported in a previous study^17^. Moreover, in the supplementary material of the study of Cao *et al*.^18^, AOA and AOB are found to be included in the total airborne archaea and bacteria identified at the genus level in PM_2.5_ and PM_10_ in Beijing during a severe smog event using metagenomic methods^18^. In the other two studies, AOB are discovered in the total bacterial communities in the total suspended particulate matter (TSP) in an urban area of Northern Italy and in the coarse particular (>3 μm) in a metropolitan subway system based on high throughput sequencing targeting 16S rRNA genes^19^,^20^. These three studies suggest the occurrence of AOA and AOB in the total archaea and bacteria in atmospheric particulates with different aerodynamic diameters using high - throughput sequencing. Based on the above arguments, ammonia compounds, AOA and AOB are present in PM_2.5_, while, the presence of Comammox in PM_2.5_ remains unknown.

The present study aimed to investigate the diversity, abundance and activity of AOMs in PM_2.5_. The detailed objectives were 1) to investigate the diversity and abundance of AOA and AOB in PM_2.5_; 2) to detect the occurrence of Comammox and to determine their abundance; 3) to estimate AOMs’ activity and contributions to ammonia oxidation in PM_2.5_; and 4) to investigate the relationships between environmental factors and AOMs. Until now, only the primer set for amplification of *Ca*. N. inopinata *amoA* gene is reported^13^, therefore, in this study, the Comammox were limited to *Ca*. N. inopinata. In this study, PM_2.5_ samples were collected in Beijing-Tianjin-Hebei (BTH) megalopolis. Diversity, abundance and activity of AOMs associated with PM_2.5_ were investigated using cloning, quantitative polymerase chain reaction (qPCR) and nitrification potential test, respectively. Multivariate statistical analyses were carried out to assess the potential impact of environmental factors on AOMs in PM_2.5_.

## Results

### Diversity of AOA and AOB in PM_2.5_

**Figure 1:**
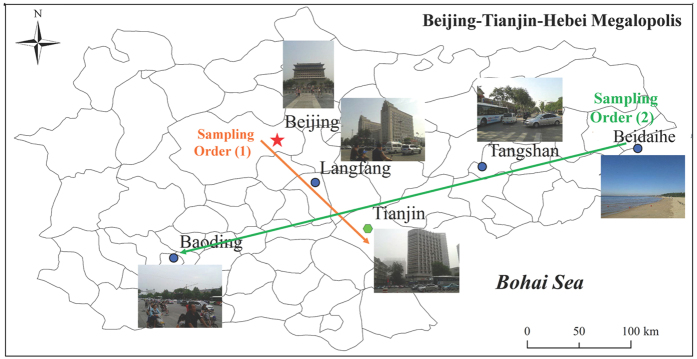
Map showing the sampling orders and sampling sites in Beijing-Tianjin-Hebei (BTH) megalopolis, China. (Figure created by the authors using MapInfo Pro v15.2 free trial, http://www.pitneybowes.com/us/miprov15-2.html and Microsoft Office 2013, https://products.office.com/zh-cn/buy/office. The photos in the figure were taken by the authors).

In this study, PM_2.5_ sampling was carried out from 21 May 2014 to 1 June 2014 at six cities in BTH, including Beijing (BJ), Langfang (LF), Tianjin (TJ), Beidaihe (BDH), Tangshan (TS) and Baoding (BD) (Fig. 1). Four types of cities were selected: two megacity sites (BJ and TJ), two industrial urban sites (TS and BD), a suburban site (LF) and a coastal site (BDH). The surroundings of sampling sites are Residents-Commercial-Transportation Mixed Districts, except BDH, which is on the coast of Bohai Sea (Fig. 1 and Table 1). Longer sampling time (24 h for each site) was used to minimize the impact of unreplicated sampling design and to account for atmospheric movements.

To investigate the presence of AOA and AOB in PM_2.5_, the specific primer sets targeting AOA *amoA* gene and AOB 16S rRNA gene were applied for PCR amplification. For all the six samples, desired single bands of PCR products were observed, which were subsequently purified and cloned. Sequencing results further confirmed that the genes were AOA *amoA* gene and AOB 16S rRNA gene, suggesting the presence of AOA and AOB in PM_2.5_ in BTH.

**Figure 2:**
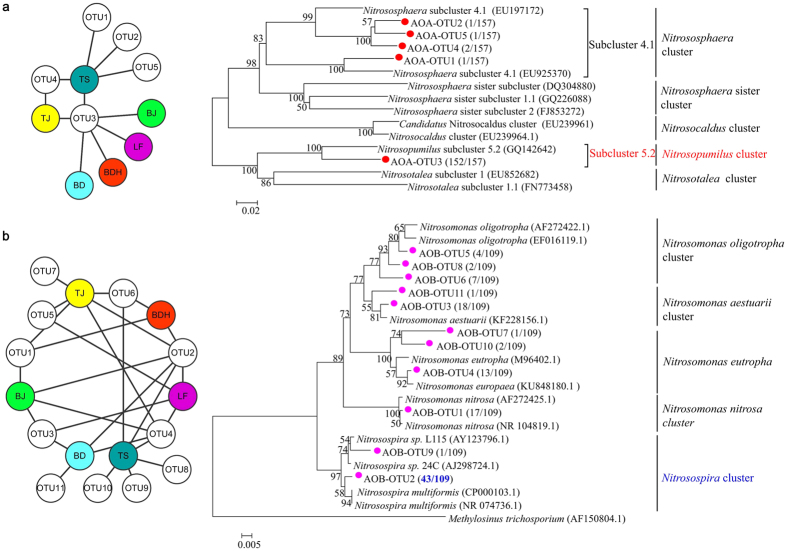
Distributions of OTUs and NJ phylogenetic tree: (**a**) AOA *amoA* gene; (**b**) AOB 16S rRNA gene. Sequences retrieved in this study are shown in red and purple with “OTU” in the names. The ratios of amounts of sequences within each OTU to the total AOA or AOB sequences are displayed by the number in parentheses.

A total of 157 AOA *amoA* gene sequences were retrieved, and five unique operational taxonomic units (OTUs) were observed at 97% sequence similarity. As shown in Fig. 2a, within each individual clone library, 1–5 OTUs occurred. OTU3 was omnipresent, occurring at all the six PM_2.5_ samples (Fig. 2a). The diversity of AOA in PM_2.5_ was low based on the diversity indexes (see Supplementary Table S1). Phylogenetic analyses showed that the five OTUs fell into *Nitrosopumilus* cluster and *Nitrososphaera* cluster (Fig. 2a). Specifically, the dominant and omnipresent OTU3 (152 of 157 sequences, 96.82%) was affiliated to *Nitrosopumilus* subcluster 5.2, indicating its widely distribution in PM_2.5_ samples. The other four OTUs (5 of 157 sequences, 3.18%) fell into *Nitrososphaera* subcluster 4.1. For sample BJ, LF, BDH and BD, *Nitrosopumilus* subcluster 5.2 was the only phylogenetic AOA group. *Nitrosopumilus* subcluster 5.2 and *Nitrososphaera* subcluster 4.1 coexisted in sample TS and TJ.

For six PM_2.5_ samples, a total of 109 AOB 16S rRNA gene sequences were analyzed. 11 OTUs were recovered at 97% sequence similarity, which were higher than those of AOA *amoA* gene. The diversity indexes further suggest higher diversity of AOB than AOA (see Supplementary Table S1). As depicted in Fig. 2b, there were 2–6 OTUs in each sample. OTU2 was shared by all the samples. OTU4 occurred at five cities and the other OTUs only appeared in one to three samples. Phylogenetic analysis revealed the co-occurrence of *Nitrsomonas* and *Nitrosospira* in PM_2.5_. Nine OTUs (65 sequences, 59.63%) were affiliated to *Nitrsomonas* cluster, including *Nitrosomonas aestuarii* (17.43%), *Nitrosomonas nitrosa* (15.60%), *Nitrosomonas eutropha* (14.68%) and *Nitrosomonas oligotropha* (11.93%). Two OTUs (44 sequences, 40.37%) fell into *Nitrosospira* cluster. Especially, the predominant OTU2 was affiliated to *Nitrosospira multiformis*, accounting for 39.45% of total AOB 16S rRNA sequences. These results suggest that *N. multiformis* and *N. aestuarii* were the most dominant AOB in PM_2.5_ in BTH.

### Abundance of AOA and AOB in PM_2.5_

**Figure 3:**
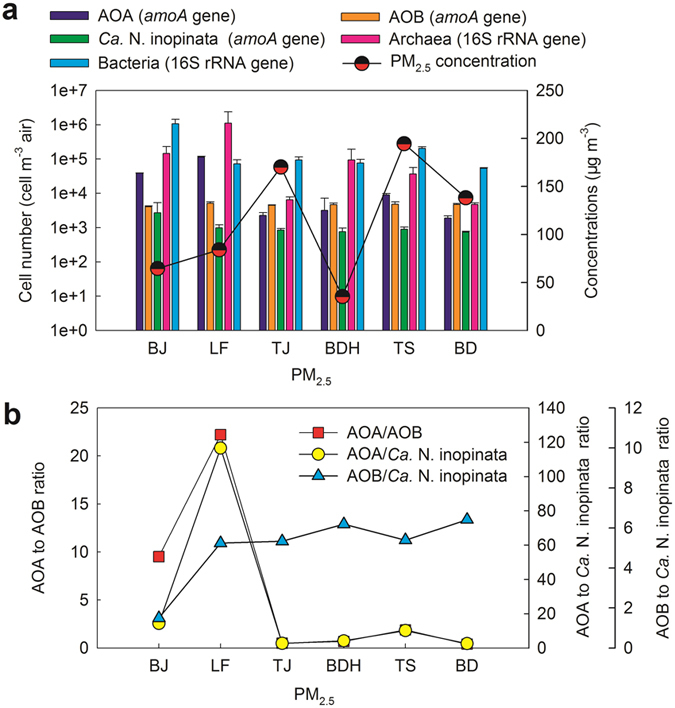
Concentrations of PM_2.5_ and quantitative analysis of AOA, AOB and *Ca.* N. inopinata in PM_2.5_: (**a**) concentrations of PM_2.5_, and abundance of AOA *amoA* gene, AOB 16S rRNA gene and *Ca.* N. inopinata *amoA* gene in PM_2.5_; (**b**) ratios of AOA to AOB, AOA to *Ca.* N. inopinata and AOB to *Ca.* N. inopinata. *Ca.* N. inopinata is a Comammox enrichment culture.

The qPCR results of AOA and AOB in PM_2.5_ are shown in Fig. 3a. The abundance of AOA ranged from 1.89 × 10^3^ ± 3.10 × 10^2^ (BD) to 1.14 × 10^5^ ± 5.31 × 10^3^ cell m^−3^ air (LF), averaged 2.82 × 10^4^ ± 1.94 × 10^3^ cell m^−3^ air. The abundance of AOA was higher in BJ and LF then in the other four cities. The abundance of AOB of PM_2.5_ in BTH was in the same order of magnitude, and the average abundance of them was 4.65 × 10^3^ ± 4.20 × 10^2^ cell m^−3^ air. The abundance of AOA was higher than that of AOB in BJ, LF and TS with the ratio of AOA to AOB ranging from 1.88 to 22.22 (Fig. 3b), while the abundance of AOA was lower than AOB in the other three cities with the ratio ranging from 0.40 to 0.68.

In order to investigate the proportions of AOA and AOB to total archaea and bacteria in PM_2.5_, abundances of archaea and bacteria were further investigated (Fig. 3a). Assuming that there are 3.6 copies of 16S rRNA gene per average prokaryotic cell^21^, the abundance of archaea and bacteria was in the range of 4.72 × 10^3^ ± 5.13 × 10^2^ (BD) to 1.10 × 10^6^ ± 1.28 × 10^6^ (LF) cell m^−3^ air and 5.36 × 10^4^ ± 1.75 × 10^3^ (BD) to 1.06 × 10^6^ ± 3.85 × 10^5^ (BJ) cell m^−3^ air, respectively. The proportions of AOA to archaea ranged from 3.38% (BDH) to 40.12% (BJ) with the average of 23.34%. The proportions of AOB to bacteria were in the range of 0.38% (BJ) to 8.79% (BD).

Moreover, as shown in Fig. 3a, concentrations of PM_2.5_ varied greatly (35.42 μg m^−3^ in BDH to 194.44 μg m^−3^ in TS) at six cities in BTH, which were higher in TJ, TS and BD than BJ, LF and BDH. However, the abundances of AOA, AOB, archaea and bacteria were relatively higher in the latter three cities, suggesting that higher concentrations of PM_2.5_ did not mean higher abundance of microorganisms. Cities TS and BD are the industrial urban sites, the emissions of toxic substances into atmosphere by industrial processes might be much higher than those in BJ, LF and BDH, which might be harmful for the survival of microorganisms, resulting in the relative low abundances.

### Presence and abundance of *Ca.* N. inopinata in PM_2.5_

The specific primer set targeting *Ca.* N. inopinata *amoA* gene was applied to investigate its presence and abundance in PM_2.5_. The expected single bands of PCR products were observed for each PM_2.5_ sample. The PCR products were purified, cloned and sequenced. In total, 63 sequences were obtained, which were further aligned with MEGA 5.0 software, and compared with those in the database of National Center for the Biotechnology Information. The results suggest that all the sequences showed 100% similarity with *Ca.* N. inopinata *amoA* gene, a Comammox enrichment culture, suggesting the presence of Comammox in PM_2.5_.

Figure 3a depicts the qPCR results of *Ca.* N. inopinata *amoA* gene in PM_2.5_. The abundance of *Ca.* N. inopinata *amoA* gene showed slight variations among six samples in BTH. The highest abundance of *Ca.* N. inopinata was observed in BJ (2.71 × 10^3^ ± 2.62 × 10^3^ cell m^−3^ air), and lowest abundance occurred in BD (7.35 × 10^2^ ± 4.53 × 10^1^ cell m^−3^ air). Compared with AOA and AOB, the abundance of *Ca.* N. inopinata was low. The ratios of AOA and AOB to *Ca.* N. inopinata ranged from 2.58 to 116.57 and 1.41 to 6.46, respectively (Fig. 3b).

### Nitrification potential of PM_2.5_

**Figure 4:**
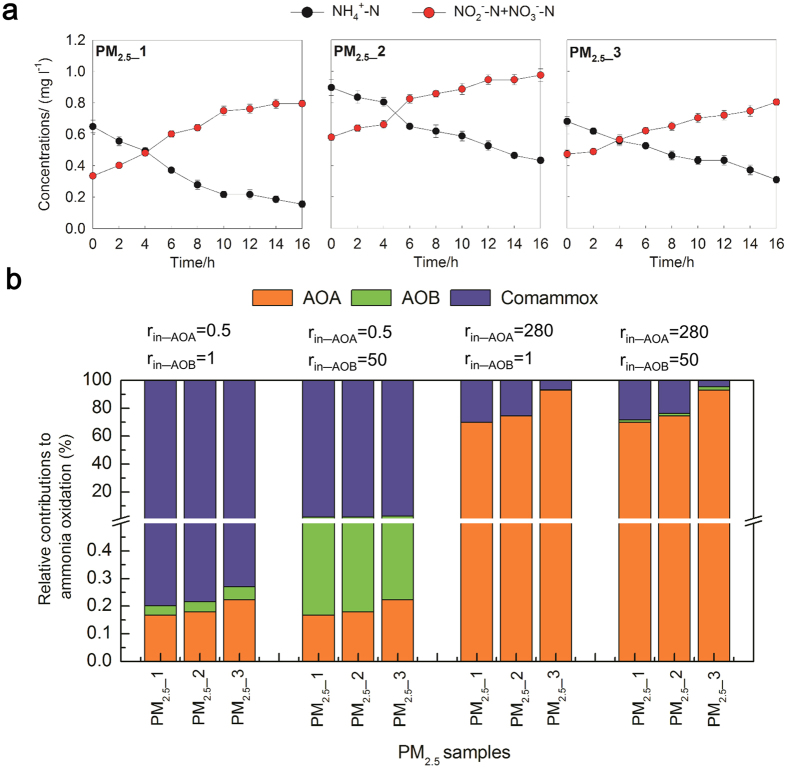
Nitrification potential test of PM_2.5_ and estimation of AOA, AOB and Comammox contributions to ammonia oxidation: (**a**) changes in net ammonia (NH_4_^+^-N) and nitrite (NO_2_^−^-N) plus nitrate (NO_3_^−^-N) during 16 h incubation of the nitrification potential test; (**b**) relative contributions of AOA, AOB and Comammox to ammonia oxidation estimated based on the *in situ* cell-specific ammonia oxidation activity (r_in_) for AOA and AOB are set at 0.5 or 280 fmol cell^−1^ h^−1^ and 1 or 50 fmol cell^−1^ h^−1^.

The nitrification potential of PM_2.5_ was evaluated by incubating PM_2.5_ suspensions with inorganic medium for 16 h. Changes in net ammonia and nitrite plus nitrate concentrations during the incubations for PM_2.5__1, PM_2.5__2 and PM_2.5__3 are depicted in Fig. 4a. The average net concentrations of NH_4_^+^-N were low throughout the incubation period, decreasing from an initial value of 0.65 mg l^−1^ to 0.15 mg l^−1^, 0.90 mg l^−1^ to 0.43 mg l^−1^ and 0.68 mg l^−1^ to 0.31 mg l^−1^ for PM_2.5__1, PM_2.5__2 and PM_2.5__3, respectively. Despite the dissimilar net concentrations of NH_4_^+^-N, the maximum nitrification rates were close, which were 0.16 μg (NH_4_^+^-N) [m^3^ air·h]^−1^ (PM_2.5__1), 0.15 μg (NH_4_^+^-N) [m^3^ air·h]^−1^ (PM_2.5__2) and 0.12 μg (NH_4_^+^-N) [m^3^ air·h]^−1^ (PM_2.5__3), respectively, averaged with 0.14 μg (NH_4_^+^-N) [m^3^ air·h]^−1^. Moreover, the decrease in ammonia concentrations was largely consistent with increase in nitrite plus nitrate concentrations, suggesting ammonia and nitrite plus nitrate balanced.

### Correlations between environmental factors and community and abundance of AOMs

**Figure 5:**
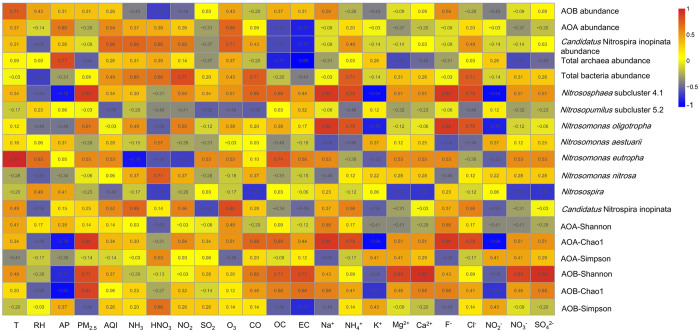
Heatmap analysis of the relationships between 23 environmental factors and diversity and abundance of AOA, AOB and *Ca.* N. inopinata based on SRCCs. SRCCs between −1 and 1 are shown in the rectangle.

Spearmans’ rank correlation coefficients (SRCCs) were calculated to investigate the relationships between environmental factors (Table 1 and Supplementary Table S2) and diversity and abundance of AOMs. The results are shown in Fig. 5. Only *Nitrososphaera* subcluster 4.1 was positively correlated with PM_2.5,_ Na^+^ and F^−^ (SRCC = 0.845, *p* = 0.034 for the three factors), and negatively correlated with K^+^ and NO_2_^−^ (SRCC = −0.845, *p* = 0.034 for the two factors). However, no significant correlations were observed between environmental factors and *Nitrosopumilus* subcluster 5.2. *N. eutropha* showed positive correlation with T (SRCC = 0.949, *p* = 0.014). *N. oligotropha* was positively correlated with Na^+^ and F^−^ (SRCC = 0.841, *p* = 0.036 for the two factors). Chao 1 of AOA was positively correlated with PM2.5, Na^+^ and F^−^ (SRCC = 0.845, *p* = 0.034 for the three factors), and negatively correlated with K^+^ and NO_2_^−^ (SRCC = −0.845, *p* = 0.034 for the two factors). Shannon index of Beta-AOB was positively correlated with Mg^2+^ (SRCC = 0.829, *p* = 0.042), NO_3_^−^ (SRCC = 0.829, *p* = 0.042), Ca^2+^ (SRCC = 0.886, *p* = 0.019) and SO_4_^2−^ (SRCC = 0.886, *p* = 0.019). Chao 1 of Beta-AOB was negatively correlated with AP (SRCC = −0.878, *p* = 0.021) and positively correlated with PM_2.5_ (SRCC = 0.812, *p* = 0.05). However, no significant correlations were observed between environmental factors and the abundance of AOMs.

**Figure 6:**
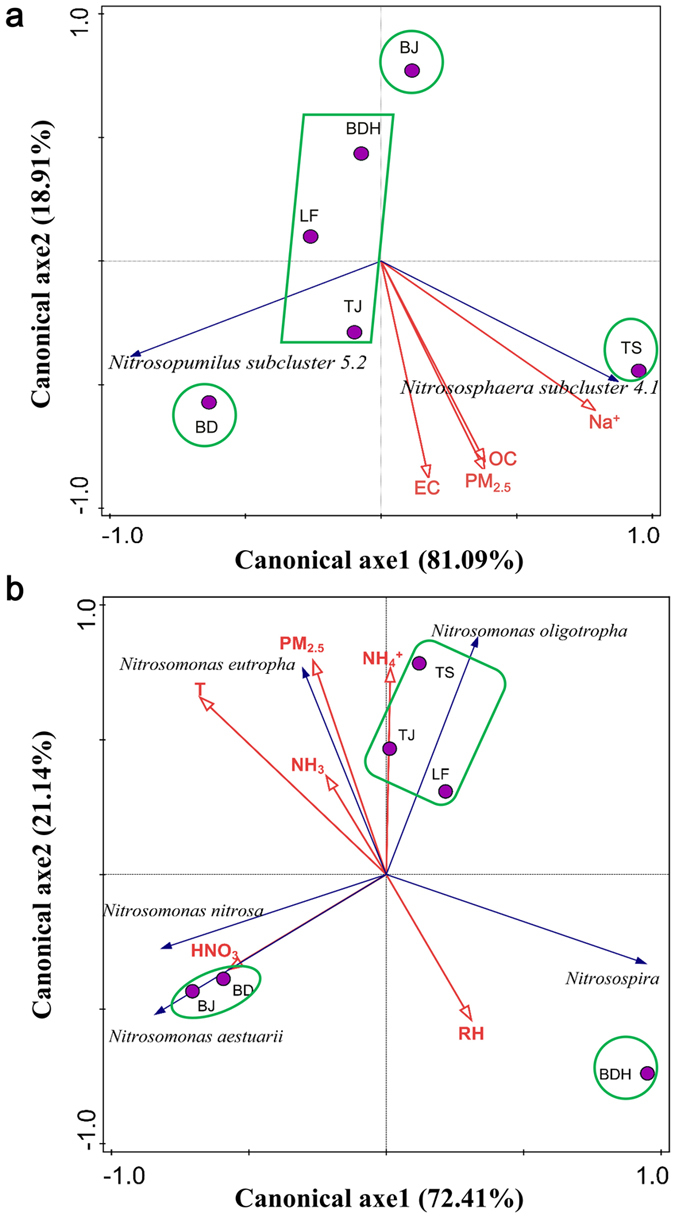
PCA and RDA ordination plots for the first two principal dimensions of the relationship between AOA and AOB community compositions and environmental factors: (**a**) AOA; (**b**) AOB.

The results of principal components analysis (PCA) and redundancy analysis (RDA) for AOA and Beta-AOB are shown in Fig. 6. As shown in Fig. 6a, the principal component 1 (PC1) and PC2 explained 81.09% and 18.91% of the variance in overall community structure, respectively. Cities BDH, LF and TJ were located close to each other, indicating the similar occurrence of AOA species. However, AOA genera in BJ, BD and TS might be different from others. RDA analysis was carried out to further explore relationships between four factors selected by Monte Carlo permutation tests and the dominant AOA genera (Fig. 6a). The results showed that *Nitrososphaera* subcluster 4.1 had positive correlations with Na^+^, PM_2.5_, organic carbon (OC) and element carbon (EC). However, *Nitrosopumilus* subcluster 5.2 was negatively related with these environmental factors. OC showed significant positive correlation with *Nitrososphaera* subcluster 4.1.

For Beta-AOB species, PC1and PC2 explained 72.41% and 21.14% of the variance in overall community structure, respectively (Fig. 6b). Three groups of six PM_2.5_ samples could be plotted off: the first group contained LF, TJ and TS, the second group contained BJ and BD, and there was only one sample (BDH) in the third group. The selected environmental factors were further used for RDA analysis (Fig. 6b). The results suggest that PM_2.5_, NH_3_, T and NH_4_^+^ showed positive correlations with *N. eutropha*. NH_4_^+^ was also positively correlated with *N. oligotropha*. HNO_3_ showed significant positive correlation with *N. aestuarii* and *N. nitrosa*. RH was positively correlated with *Nitrosospria*.

## Discussion

AOA or AOB are present in particulate matter with different aerodynamic diameters, e.g., PM_2.5_ and PM_10_ in Beijing^18^, coarse particulate (>3 μm) in Germany^17^, TSP in an urban area of Northern Italy^19^, and coarse particular (>3 μm) in a metropolitan subway system of New York^20^. This study provided fundamental information regarding AOA, AOB and Comammox in PM_2.5_ in BTH. The newly discovered Comammox are widely distributed in a variety of environments, including natural and man-made ecosystems^6^,^13^,^14^,^22^. In this study, the occurrence of *Ca.* N. inopinata, a Comammox enrichment culture, in PM_2.5_ expanded our knowledge of nitrification. The diversity of AOA and AOB was scarce in PM_2.5_, which might be due to the extremely low level of nutrient in the atmosphere^23^.

In China, the total ammonia emission was 16.55 Tg for 2005 and keeps an increase trend^24^. Ammonia plays a significant role in the neutralization of acid species to form SIA and PM_2.5_ pollution^4^. Ammonia is also the energy source for AOA and AOB, and plays an important role in the niche separations of different species of them^16^. In this study, *Nitrosopumilus* subcluster 5.2, was found to be the dominant AOA species (96.82%) in PM_2.5_, and *Nitrososphaera* cluster only accounted for a small percentage (3.18%). *Nitrosopumilus maritimus*, the cultivated representative of *Nitrosopumilus*, possesses high affinity to ammonia with low half-saturation constant (*K*_m_ = 0.133 μmol l^−1^)^16^. In this study, the low ammonia concentrations in the atmosphere (0.363–0.947 mg m^−3^) might be a reason for the dominance of *Nitrosopumilus*. Also, *Nitrosopumilus* is a marine AOA clade^16^, indicating that AOA observed in PM_2.5_ might be similar to those in marine. Furthermore, the air movement might make *Nitrosopumilus* cluster prevail in a broader area, not only in BTH.

The previous studies suggest that *Nitrosomonas* and *Nitrosospira* were the main AOB in particulate matters^18^,^19^,^20^, which was in agreement with our study. In this study, 59.63% of AOB in PM_2.5_ fell into *Nitrosomonas* cluster and 40.37% of them fell into *Nitrosospira* cluster. *N. multiformis* and *N. aestuarii* were the most dominant AOB in PM_2.5_ in BTH. *N. multiformis* is a commonly used model organism for soil AOB, as it is a representative of *Nitrosospira* cluster 3, which is widespread in agricultural soils^25^,^26^. *N. aestuarii*, one of the marine AOB species, is retrieved from marine environments^27^. These results suggest that AOB observed in PM_2.5_ might be similar to those in agricultural soils and marine. Moreover, *N. nitrosa* (15.60%), *N. eutropha* (14.68%) and *N. oligotropha* (11.93%) were also the main AOB in PM_2.5_. Members of *N. nitrosa* and *N. oligotropha* exhibit relatively low *K*_m_ values^28^. Therefore, the low ammonia concentration in the atmosphere is in favor of their survival. *N. nitrosa* and *N. eutropha* are also the main AOB in eutrophic freshwater. Members of *N. eutropha* are common in eutrophic freshwater habitats^27^.

Occurrence of *Ca.* N. inopinata in PM_2.5_ in BTH, suggesting that atmosphere (a substrate-limiting environment) maybe a common habitat for Comammox. Previous studies suggest that Comammox may have an advantage over AOA and AOB under substrate-limiting environments^6^,^14^. Low substrate is in favor of the enrichment of Comammox. van Kessel *et al*. enrich the biofilm samples collected from a recirculation aquaculture system in the presence of low ammonia concentration for one year, after which the cultures are capable of complete nitrification and are mostly composed of microorganisms belonging to Comammox *Nitrospira* bacteria^14^. Daims *et al*. enrich the biofilm samples collected from a pipe in a deep oil exploration well under low ammonia concentration for four years, after which most of the microorganisms within the culture are Comammox *Nitrospira* bacteria^13^. Overall, the low ammonia concentration in the atmosphere might be the main reason for occurrence and ecological niche distribution of AOMs in PM_2.5_.

Relationships between environmental factors and the communities of AOA and AOB were further investigated by SRCC, PCA and RDA. Some interesting relationships were observed. OC showed significant positive correlation with *Nitrososphaera* subcluster 4.1, suggesting that some compounds of OC could stimulate their growth. Kim *et al*.^29^ has confirmed the activity of AOA strain DDS1 isolated from seawater can be enhanced by adding α-keto acids (e.g., pyruvate, oxaloacetate). These organic carbon substrates are not assimilated as a carbon source but act as chemical scavengers, suggesting that AOA broadly feature strict autotrophic nutrition^29^. RDA results also indicate that NH_3_ showed positive correlation with *N. eutropha* cluster. Previous studies suggest that high ammonia concentration is in favor of *N. eutropha* cluster^16^,^30^, which was in accordance with our study.

The average abundance of AOA and AOB was 2.82 × 10^4^ and 4.65 × 10^3^ cell m^−3^ air, respectively. Comparisons of AOA and AOB cell numbers of PM_2.5_ with different types of samples (soils, compost samples, activated sludge samples and sediment samples) from previous studies are summarized in Supplementary Table S3. AOA abundance in PM_2.5_ was close to the quantities of AOA reported for soils collected from a large geographical scale across North to South China with different pH values^31^, activated sludge treating domestic wastewater^32^ and sediments in the hyporheic zone of a eutrophic river in North China^33^. The abundance of AOB in PM_2.5_ was comparable with the compost samples collected from the suburb of Changsha, China^34^ and the activated sludge samples treating municipal wastewater^35^. The average abundance of AOA was one order of magnitude higher than that of AOB, which was consistent with most of the studies mentioned in Supplementary Table S3. The average abundance of *Ca.* N. inopinata was 1.15 × 10^3^ cell m^−3^ air, which was in the same order of magnitude with AOB, but one to two orders of magnitude lower than AOA. However, the abundance of total Comammox in PM_2.5_ is still unknown. Further investigations are needed to investigate the abundance of Comammox in PM_2.5_ with suitable primer set.

Thus far, the actual contributions of AOA, AOB and Comammox to ammonia oxidation in PM_2.5_ remain unknown. The maximum nitrification rate (NNR_max), *in situ* cell-specific ammonia oxidation activity (r_in_, fmol cell^−1^ h^−1^) for AOA and AOB, and the abundances of AOA and AOB were used to estimate their relative contributions to ammonia oxidation with formulas (3), (4) and (5) mentioned in methods. Ammonia assimilation of heterotrophic bacteria could remove 10–30% of the ammonia^36^,^37^,^38^. However, in this study, the ammonia assimilation of heterotrophic bacteria was not considered in the calculation of relative contributions of AOMs to nitrification because the ammonia assimilation was insignificant in low ammonia concentration environment^39^. On the one hand, in direct nutrient-limited competition, the ammonium turnover per unit biomass of *Nitrosopumilus*-like AOA would be at least 5 times higher than of oligotrophic heterotrophs^16^. On the other hand, the half-saturation constant (*K*_m_) of *Nitrosopumilus* cluster for ammonia is much lower than the lowest *K*_m_ of ammonia assimilation of heterotrophic bacteria^16^.

In the present study, the r_in_ for AOA was set at 0.5 or 208 fmol cell^−1^ h^−1^ by referring to the following studies on *in situ* activity of AOA: 0.5 fmol cell^−1^ h^−1^ in freshwater sediment^40^, 0.53 fmol cell^−1^ h^−1^ for *N. maritimus* SCM1^16^, 4.9–80.6 in drinking water treatment processes^39^, and 0.03–207.97 fmol cell^−1^ h^−1^ in WWTPs^41^. For AOB, the r_in_ was set at 1 or 50 fmol cell^−1^ h^−1^ according to the following studies reported the r_in_ values: 4.4–23.0 fmol cell^−1^ h^−1^ for AOB isolates^42^,^43^, 1.3–8 fmol cell^−1^ h^−1^ in freshwater sediment^44^; and 0–49.6 fmol cell^−1^ h^−1^ in WWTPs^45^.

Figure 4b shows the estimations of relative contributions of AOA, AOB and Comammox to ammonia oxidation based on different r_in_ for AOA and AOB. If r_in_ for AOA and AOB were set as 0.5 and 1 fmol cell^−1^ h^−1^ or 0.5 and 50 fmol cell^−1^ h^−1^, the relative contributions of AOA and AOB to ammonia oxidation were extremely low, and Comammox accounted for almost 100% of ammonia oxidation for the three PM_2.5_ samples. Since the ammonia concentrations in the nitrification potential test were low and AOA have lower *K*_m_ and higher affinity to ammonia^16^, AOA contribution in the test may be underestimated. If r_in_ values for AOA and AOB were set as 208 and 1 fmol cell^−1^ h^−1^, for three PM_2.5_ samples, AOA, AOB and Comammox were responsible for 69.83–93.10%, 0.04–0.05% and 6.85–30.14% of the ammonia oxidation, respectively. If r_in_ values for AOA and AOB were set as 208 and 50 fmol cell^−1^ h^−1^, AOA, AOB and Comammox were responsible for 69.83–93.10%, 1.76–2.35% and 4.55–28.41% of the ammonia oxidation, respectively. These two results suggest that AOA accounted for most of the ammonia oxidation, followed by Comammox, however, the contribution of AOB to ammonia oxidation was low, which might be related to the low ammonia concentration in PM_2.5_ incubations and the higher abundance of AOA and Comammox in PM_2.5_. In previous studies, the same calculation methods were used to evaluate the relative contributions of AOA and AOB to the nitrification of activated sludge in full-scale WWTPs^41^ and granular activated carbon used in a full-scale advanced drinking water treatment plant^39^. Their results reveal the significant contribution made by AOA to nitrification under low ammonia concentrations, which were consistent with our study. While, their results also suggest AOB play the dominant role of nitrification under higher ammonia concentration conditions^41^. In fact, a more accurate estimation of their contributions to nitrification should only depend on the active AOMs. RNA-based methods or DNA based stable-isotope probing (DNA-SIP) technique may be more effective and accurate to evaluate the contributions of AOMs to nitrification. Further investigation is still needed to validate the hypothesis that AOA played the predominant role in ammonia oxidation of PM_2.5_.

In conclusion, *Nitrosopumilus* subcluster 5.2 was the most dominant AOA. *N. multiformis* and *N. aestuarii* were the main AOB. The occurrence of Comammox in PM_2.5_ was confirmed by the presence of *Ca.* N. inopinata in PM_2.5_. The average cell numbers of AOA, AOB and *Ca.* N. inopinata were 2.82 × 10^4^, 4.65 × 10^3^ and 1.15 × 10^3^ cell m^−3^ air, respectively. *Nitrososphaera* subcluster 4.1 was positively correlated with PM_2.5_ and OC concentrations, and *N. eutropha* cluster and *N.* aestuarii cluster showed positive correlations with NH_3_ and HNO_3_ concentrations. The average maximum nitrification rate of PM_2.5_ was 0.14 μg (NH_4_^+^-N) [m^3^ air·h]^−1^. AOA and Comammox may be the major contributors to ammonia oxidation in PM_2.5_. However, further investigations regarding Comammox in PM_2.5_ based on an appropriate primer set are still needed.

## Methods

### Sample collection, meteorological conditions and chemical analyses

A model KC-6120 comprehensive atmospheric sampler (Laoshan Electronic Instrument Factory, Qingdao, China) was used for the collection of PM_2.5_, NH_3_ and HNO_3_ samples. The glass fiber filters were pre-heated at 450 °C for 4 h to remove organic material and their weight were measured by a microbalance before PM_2.5_ collection. PM_2.5_ collection was carried out at a flow rate of 100 l min^−1^ for 24 h (Table 1). The collections of NH_3_ and HNO_3_ were according to the national standard method of the People’s Republic of China (GB/T 18204.25 2000). During summer, the predominant wind direction of BTH is from southeast; therefore, as shown in Fig. 1, the first order of samples collection was from BJ to LF and then to TJ. After sampling from the three cities, samples were collected from the northeast to southwest axis of BTH (namely BDH-TS-BD).

The meteorological data, atmospheric pollutants and air pollution index (AQI) were recorded concurrently with air sampling (Table 1 and Supplementary Table S2). The carbonaceous species (OC and EC) and water-soluble inorganic ions of PM_2.5_ were analyzed by a thermal/optical carbon aerosol analyzer (DRI Model 2001A, Desert Research Institute, USA) and ion chromatography (ICS-90, Dionex, USA), respectively (see Supplementary Table S2).

### DNA extraction, PCR, cloning and sequencing

For PM_2.5_ samples, 1/4 of the whole glass fiber was cut into pieces using sterilized handling instruments. Genomic DNA was extracted using a Fast-DNA ^®^ SPIN Kit following the manufacturer’s protocol (Qiagen, CA, USA).

Primer sets Arch-amoAF/Arch-amoAR^46^, CTO189f/CTO645r^47^, amoA-3F/amoB-4R^48^ and Nino_amoA_19F/Nino_amoA_252R^13^ were used to amplify AOA *amoA* gene, Beta-AOB 16S rRNA gene, Gamma-AOB *amoA* gene and *Ca.* N. inopinata *amoA* gene fragments of PM_2.5_ samples. The CTO189f was a mixture of CTO189fA/B and CTO189fc at a ratio of 2:1. For AOA *amoA* gene, the components of PCR mixture and protocols of PCR were followed by the study of Gao *et al*.^35^. For Beta-AOB 16S rRNA gene and *Ca.* N. inopinata *amoA* gene, the PCR protocols were the same as AOA *amoA* gene except the annealing temperature (58 °C and 60 °C for Beta- AOB and *Ca.* N. inopinata, respectively). Gradient PCR was applied to detect Gamma-AOB in PM_2.5_. However, the amplification of Gamma-AOB *amoA* gene was failed. PCR products for the other genes were purified, cloned and sequenced. For each sample, 15–30 white colonies for AOA *amoA* gene, Beta-AOB 16S rRNA gene and *Ca.* N. inopinata *amoA* gene were randomly picked for sequencing with ABI 3730 XL capillary sequencers (PE Applied Biosystems, Foster City, USA).

The AOA *amoA* gene, Beta-AOB 16S rRNA gene and Ca. N. inopinata *amoA* gene sequences have been deposited in the GenBank library under accession numbers from KM402456 to KM402612, KY008589 to KY008697 and KX273257 to KX273319, respectively.

### Phylogenetic analyses

The sequences were grouped into OTUs with a 97% sequence similarity using Mothur 1.28. Cytoscape 2.32 was applied for visualization of the shared OTUs between samples. MEGA 5.0 was used to construct a phylogenetic tree using the neighbor-joining (NJ) method with the Jukes–Cantor correction model. The NJ tree was calculated after bootstrapping with 1000 replicate trees.

### Quantification of AOA, AOB, Comammox, bacteria and archaea of PM_2.5_

The abundance of AOA, AOB, *Ca.* N. inopinata, bacteria and archaea of PM_2.5_ were quantified by the following primer sets: GenAOAF/GenAOAF^49^, amoA-1Fmod and GenAOBR^49^, Nino_amoA_19F/Nino_amoA_252R^13^, Uni1055F and 1392R^50^ and 934f/1040r^51^ on a Stratagene MX3005p thermocycler (Agilent Technologies, USA) in triplicate with a GoTaq^®^ qPCR Master Mix (Promega, USA). The components of qPCR mixture were the same as the previous study^35^. The qPCR conditions were also followed by this study except different annealing temperature: 56 °C, 58 °C, 60 °C, 53 °C and 59 °C for AOA, AOB, *Ca.* N. inopinata, bacteria and archaea. The standard curve was generated by using 10-fold serial dilutions of linearized plasmid extracted from the correct insert clones of each target gene. The amplification efficiencies of qPCR assays ranged from 90.6 to 106.0%, and *R*^*2*^ value for each standard curves exceeded 0.996.

### Nitrification potential test

In order to investigate the nitrification potential of PM_2.5_, three PM_2.5_ samples (PM_2.5__1, PM_2.5__2 and PM_2.5__3) were collected in BJ for 24 h (8 h for each sampling) at a flow rate of 100 l min^−1^, resulting a total of 48 m^3^ air collected for each sample. A blank filter (as control) was put at the side of the sampler with each PM_2.5_ sampling, therefore three blank samples (Blnak_1, Blank_2 and Blank_3) were obtained. After sampling, the PM_2.5_ and blank filters were immediately wrapped with aluminum foil and taken back to the lab within five minutes.

Technologies for investigation of microorganisms in the atmosphere are still in immature stage of development, and the methods for evaluation of nitrification activity of AOMs in PM_2.5_ have not been reported. The overall microbial community composition in PM_2.5_ maybe similar as that in soil because soil is one of the main source for PM_2.5_^52^. Soil suspension technique is a recommended method for assessing nitrification potential^53^. In this technique, the substrate and moisture limitations are eliminated, and the changes in AOMs are unlikely to occur after short-term incubation. Therefore, the nitrification rate measured approximates the maximum nitrification rate possible at the specific temperature of the incubation^53^. Moreover, this technique may be the easiest to interpret and most reproducible for all laboratory nitrification assays^53^.

In this study, maximum nitrification rate of PM_2.5_ was investigated according to soil suspension technique with some modification, which may be defined as PM_2.5_ suspension technique. Briefly, the PM_2.5_ and blank filters were cut into pieces and put into 50 ml centrifuge tubes filled with sterilized and oxygenated phosphate-buffered saline (PBS) (g l^−1^: NaCl, 8.0; KCl, 0.2; Na_2_HPO_4_, 1.44; KH_2_PO_4_, 0.24; pH, 7.4) followed by vortexing for 30 min and sonication for 2 h to generate suspensions of PM_2.5_ and blank samples. The 50 ml suspensions of PM_2.5_ and blank samples were incubated individually with 200 ml of inorganic medium in 500 ml Erlenmeyer flasks. The Erlenmeyer flasks were closed with plastic wrap and incubated at 30 °C under agitation at 100 rpm. Compositions of inorganic medium were as follows: 3 ml NH_4_Cl (10 μg l^−1^), 3 ml NaHCO_3_ (20 μg l^−1^), 0.25 ml trace element and 193.75 ml PBS. The compositions of trace element were according to the previous study^54^. After 0, 2, 4, 6, 8, 10, 12, 14, 16 h of incubation, supernatant was collected and filtered through 0.2 μm pore size polytetrafluoroethylene membranes. Concentrations of ammonia (NH_4_^+^-N), nitrite (NO_2_^−^-N) and nitrate (NO_3_^−^-N) were analyzed in triplicate in accordance with standard methods^55^.

The net concentrations of NH_4_^+^-N (NH_4_^+^-N_net, mg l^−1^) in PM_2.5_ sample incubations were calculated using the following formula:


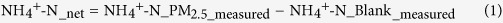


where NH_4_^+^-N_PM_2.5_measured_ and NH_4_^+^-N_Blank__measured_ are the measured ammonia concentration in the incubations of PM_2.5_ and blank samples.

The calculations of net concentrations of NO_2_^−^-N and NO_3_^−^-N in PM_2.5_ sample incubations were the same as the NH_4_^+^-N. The NNR_max (μg (NH_4_^+^-N) [m^3^ air·h]^−1^) of PM_2.5_ samples were calculated by the following formula:


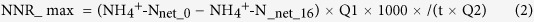


where NH_4_^+^-N _net_0_ and NH_4_^+^-N _net_16_ are the net ammonia concentrations in PM_2.5_ incubations at 0 and 16 h, Q1 is the incubation volume (Q1 = 0.25 l), t is the incubation time (t = 16 h), and Q2 is the volume of sampled air (Q2 = 48 m^3^).

### Estimation of relative contributions of AOA, AOB and Comammox to ammonia oxidation in PM_2.5_

The estimation of relative contributions of AOA, AOB and Comammox to ammonia oxidation was carried out with some assumptions: (1) only AOA, AOB and Comammox determined were involved in the ammonia oxidation in PM_2.5_; (2) the ammonia assimilation of heterotrophic bacteria was not considered; (3) there were 1 *amoA* gene copy per AOA and Comammox, and 2.5 *amoA* gene copies per AOB^13^,^15^,^21^; (4) all AOA, AOB and Comammox were equally active enough to contribute to ammonia oxidation. Their relative contributions (RC) to ammonia oxidation of PM_2.5_ were estimated using the following formulas according to previous studies^36^,^39^,^41^:


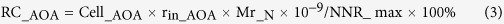



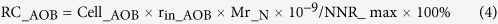



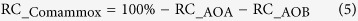


where RC__AOA_, RC__AOB_ and RC__Comammox_ represent the relative contribution of AOA, AOB and Comammox. Cell__AOA_ and Cell__AOB_ are the abundance of AOA and AOB (cells m^−3^ air), and r_in___AOA_ and r_in___AOB_ are the *in situ* cell-specific ammonia oxidation activity (r_in_, fmol cell^−1^ h^−1^) for AOA and AOB. Mr__N_ is the relative molecular mass of nitrogen. NNR_max is the maximum nitrification rate (μg (NH_4_^+^-N) [m^3^ air·h]^−1^).

### Statistical analysis

SRCC, PCA and RDA were applied to address the correlations between environmental factors and AOMs (AOA, Beta-AOB and *Ca.* N. inopinata). The Monte Carlo permutation test (999 replicates) was used to estimate the significance of the correlations. All of the statistical analyses were done using R software version 2.15.

## Additional Information

**How to cite this article**: Gao, J.-F. *et al*. Diversity, abundance and activity of ammonia-oxidizing microorganisms in fine particulate matter. *Sci. Rep.* **6**, 38785; doi: 10.1038/srep38785 (2016).

**Publisher's note:** Springer Nature remains neutral with regard to jurisdictional claims in published maps and institutional affiliations.

## Supplementary Material

Supplementary Material

## Figures and Tables

**Table 1:** Description of geography information of sampling sites, samples collected and corresponding meteorological conditions in BTH.

Cities	City type	Longitude and latitude of locations	Sampling date*		PM_2.5_ (μg m^−3^)	NH_3_ (μg m^−3^)	T (°C)	RH(%)	AP (Hpa)	AQI
			Start time	End time						
Beijing	Megacity	39°53′28″N	5/21 8:00		64.58	0.903 ± 0.140	24	52	1005.25	183
(BJ)		116°23′29″E		5/22 11:30			27	48	1006.11	188
Langfang	Semi-urban area	39°31′16″N	5/23 8:00		83.93	0.740 ± 0.068	31	34	1009.64	138
(LF)		116°43′15″E		5/24 11:30			23	75	1007.89	104
Tianjin	Megacity	39°7′14″N	5/25 8:00		170.14	0.947 ± 0.718	24	54	1006.31	134
(TJ)		117°10′40″E		5/26 11:30			26	20	1000.35	82
Beidaihe	Coastal city	39°48′58″N	5/27 8:00		35.42	0.169 ± 0.240	17	62	1008.00	79
(BDH)		119°31′12″E		5/28 11:30			19	60	1007.00	54
Tangshan	Industrial city	39°39′29″N	5/29 8:00		194.44	0.363 ± 0.412	23	51	1004.00	192
(TS)		118°10′13″E		5/30 11:30			32	54	1004.00	201
Baoding	Industrial city	38°51′58″N	5/31 8:00		138.19	0.000	30	56	1004.00	158
(BD)		115°29′32″E		6/1 11:30			23	57	1005.00	73

Abbreviations: T: Temperature; RH: Relative Humidity; AP: Atmospheric Pressure.

^*^PM_2.5_ collection was carried out at a flow rate of 100 l min^−1^ for 24 h (from 08:00 a.m. to 11:30 a.m. the next day with procedure of 3 h collection and 0.5 h interval to prevent the overheating of the pump).
