# Supplementary material for: Diversity, abundance and activity of ammonia-oxidizing microorganisms in fine particulate matter
Source: Sci Rep. 2016 Dec 12;6:38785. doi: 10.1038/srep38785 (PMC5150234; doi:10.1038/srep38785)
Supplement: Supplementary Material [file srep38785-s1.pdf]

Supplementary Material

Diversity, abundance and activity of ammonia-oxidizing microorganisms in fine particulate matter

Jing-Feng Gao *, Xiao-Yan Fan, Kai-Ling Pan, Hong-Yu Li and Li-Xin Sun

Correspondence and phone calls about the paper should be directed to Jing-Feng Gao at the following address, phone and fax number, and e-mail address:

Jing-Feng Gao

College of Environmental and Energy Engineering, Beijing University of Technology, 100 Pingleyuan, Chaoyang District, Beijing 100124, China

Tel: +86-10-6739-2627(office); Fax: +86-10-6739-1983

E-mail: gao.jingfeng@bjut.edu.cn or gao158@gmail.com

Supplementary Table S1 AOA and AOB sequence diversity and Good's coverage of the six $PM_{2.5}$ clone libraries.

Supplementary Table S2 Concentrations of OC, EC, water-soluble inorganic ions and atmospheric pollutants of six cities in BTH.

Supplementary Table S3 Comparisons of AOA and AOB *amoA* gene abundances in different types of samples from literature.

Table S1 AOA and AOB sequence diversity and Good's coverage of the six $PM_{2.5}$ clone libraries

Target gene	Sample_ID	Numbers of sequences	OTUs(3% cut-off)	Good's coverage/(%)	Chao1	OUT observed/OUT estimated(%)	Shannon index	ACE	Simpson
AOA *amoA* gene	BJ	24	1	100.00	1.00	100.00	0.00	0.00	1.00
	LF	26	1	100.00	1.00	100.00	0.68	0.00	0.49
	TJ	27	2	96.30	2.00	100.00	0.16	0.00	0.93
	BDH	25	1	96.00	1.00	100.00	0.17	0.00	0.92
	TS	27	5	85.19	11.00	45.45	0.62	0.00	0.72
	BD	28	1	100.00	1.00	100.00	0.00	0.00	1.00
AOB 16S rRNA	BJ	20	4	100.00	4.00	100.00	1.19	4.00	1.00
	LF	15	4	93.33	4.00	100.00	1.16	4.70	0.93
	TJ	18	6	90.89	7.00	85.71	1.40	6.27	0.91
	BDH	20	2	95.00	2.00	100.00	0.20	0.00	0.95
	TS	16	5	93.75	5.00	100.00	1.42	5.55	0.94
	BD	20	6	90.00	6.50	92.31	1.53	7.72	0.90

Table S2 Concentrations of OC, EC, water-soluble inorganic ions and atmospheric pollutants of six cities in BTH

Cities	OC	EC	Na^+	$NH4^+$	K^+	Mg^{2+}	Ca^{2+}	F^-	Cl^-	NO_2^-	NO_3^-	SO_4^{2-}	NO_2*	SO_2*	O_3*	CO*	HNO_3
BJ	4.07	2.97	0.00	0.1	0.01	1.16	2.76	0.00	0.11	0.01	1.05	2.69	66.45±28.15	33.04±4.95	115.16±102.51	1.92±0.38	0.193±0.154
LF	4.39	3.56	0.01	0.08	0.01	0.43	2.05	0.01	0.09	0.01	0.42	2.00	30.52±15.84	27.22±14.31	143.87±59.53	0.64±0.23	0
TJ	6.45	5.14	0.01	0.31	0.01	0.72	2.40	0.01	0.36	0.01	0.67	2.34	68.22±34.26	28.96±22.79	54.26±45.72	1.80±0.67	0
BDH	4.56	3.88	0.00	0.06	0.01	0.55	1.79	0.00	0.07	0.02	0.52	1.75	30.22±15.12	62.48±13.77	46.35±15.13	0.63±0.43	0
TS	14.47	8.61	0.03	0.23	0.00	2.1	3.82	0.03	0.27	0.00	1.86	3.72	60.48±32.28	81.17±51.68	182.87±88.58	3.30±3.08	0
BD	11.97	8.84	0.01	0.05	0.01	1.39	3.75	0.01	0.06	0.01	1.25	3.66	26.42±7.17	37.74±17.61	46.53±17.26	0.95±0.76	0

*: Concentrations of atmospheric pollutants (NO_2, SO_2, O_3 and CO) were the average of that obtained hourly from the China National Environmental Monitoring Center (http://113.108.142.147:20035/emcpublish/).
The unit of all the pollutants was μg m^{-3}, except CO (mg m^{-3}).

Table S3 Comparisons of AOA and AOB *amoA* gene abundances in different types of samples from literature.

Reference	Samples	AOA *amoA* gene	AOB *amoA* gene	AOA *amoA* gene of our study compared to the reference	AOB *amoA* gene of our study compared to the reference
1	**Sandy soils**	7.0×10^6-1.0×10^8 cell /g dry soil	2.4×10^4-2.0×10^7 cell /g dry soil	Lower (<)	Higher (>)
2	**Paddy soils**	7.7×10^6-1.8×10^7 cell/g paddy soil	9.7×10^5-2.6×10^6 cell/g paddy soil	Lower (<)	Higher (>)
3	**Soils**	2.52×10^5 - 3.36×10^8 cell/g soil	1.2×10^5 – 3.3×10^7 cell/g soil	Comparable (~)	Higher (>)
4	**Compost samples**	2.8×10^8-1.1×10^9 cell /g DW composst sample	8.8×10^7-1×10^9 cell /g DW composst sample	Lower (<)	Comparable (~)
5	**Activated sludge** (Saline sewage)	1.7×10^2 to 3.8×10^3 cell/ng DNA	1.2×10^3 to 9.2×10^4 cell/ng DNA	Lower (<)	Lower (<)
6	**Activated sludge** (Municipal wastewater)	3.07×10^5 cell/g sludge	2.9×10^7 cell/g sludge	Higher (>)	Comparable (~)
7	**Activated sludge** (Domestic wastewater)	6.0×10^5-4.5×10^6 cell/g sludge	2.9×10^3-6.8×10^4 cell/g sludge	Comparable (~)	Higher (>)
8	**Sediment** (a eutrophic river)	1.48×10^7-5.50×10^7 cell/g dry sediment	1.6×10^4-7.0×10^4 cell/g dry sediment	Comparable (~)	Higher (>)

The abundance of AOA and AOB are transformed to cell number based on that there are 1 and 2.5 *amoA* genes per AOA and AOB.

References:

1. Leininger, S. et al. Archaea predominate among ammonia-oxidizing prokaryotes in soils. *Nature* **442**, 806-809 (2006).

2. Wang, J., Wang, W. & Gu, J. Community structure and abundance of ammonia-oxidizing archaea and bacteria after conversion from soybean to rice paddy in albic soils of northeast China. *Appl. Microbiol. Biotechnol.* **98**, 2765-2778 (2014).

3. Hu, H., Zhang, L., Dai, Y., Di, H. & He, J. Ph-dependent distribution of soil ammonia oxidizers across a large geographical scale as revealed by high-throughput pyrosequencing. *J. Soil Sediment* **13**, 1439-1449 (2013).

4. Zeng, G. et al. Relative contributions of archaea and bacteria to microbial ammonia oxidation differ under different conditions during agricultural waste composting. Bioresoure Technol. **102**, 9026-9032 (2011).

5. Jin, T., Zhang, T. & Yan, Q. Characterization and quantification of ammonia-oxidizing archaea (AOA) and bacteria (AOB) in a nitrogen-removing reactor using T-RFLP and qPCR. *Appl. Microbiol. Biotechnol.* **87**, 1167-1176 (2010).

6. Gao, J., Luo, X., Wu, G., Li, T. & Peng, Y. Quantitative analyses of the composition and abundance of ammonia-oxidizing archaea and ammonia-oxidizing bacteria in eight full-scale biological wastewater treatment plants. Bioresoure Technol. **138**, 285-296 (2013).

7. Bai, Y., Sun, Q., Wen, D. & Tang, X. Abundance of ammonia-oxidizing bacteria and archaea in industrial and domestic wastewater treatment systems. *FEMS Microbiol. Ecol.* **80**, 323-330 (2012).

8. Wang, Z., Wang, Z., Huang, C. & Pei, Y. Vertical distribution of ammonia-oxidizing archaea (AOA) in the hyporheic zone of a eutrophic river in north China. *World J. Microbiol. Biotechnol*. **30**, 1335-1346 (2014).
